# Activation of multiple Eph receptors on neuronal membranes correlates with the onset of optic neuropathy

**DOI:** 10.1186/s40662-023-00359-w

**Published:** 2023-10-02

**Authors:** Thomas A. Strong, Juan Esquivel, Qikai Wang, Paul J. Ledon, Hua Wang, Gabriel Gaidosh, David Tse, Daniel Pelaez

**Affiliations:** 1 Bascom Palmer Eye Institute, Department of Ophthalmology, University of Miami Miller School of Medicine, Miami, FL USA; 2 Dr. Nasser Al-Rashid Orbital Vision Research Center, Bascom Palmer Eye Institute, University of Miami Miller School of Medicine, 1638 NW 10th Avenue, Miami, FL 33136 USA; 3 Department of Biomedical Engineering, University of Miami College of Engineering, University of Miami, Coral Gables, FL USA; 4 Department of Cell Biology, University of Miami Miller School of Medicine, Miami, USA; 5 Sylvester Comprehensive Cancer Center, University of Miami Miller School of Medicine, Miami, FL USA; 6 Department of Public Health Sciences, University of Miami Miller School of Medicine, Miami, FL USA; 7 Department of Physics, University of Florida College of Liberal Arts and Sciences, Gainesville, FL USA

**Keywords:** Optic neuropathy, Ephrin signaling, Eph receptor, Axonal guidance, Neuropathy, Neurodegeneration, Optic nerve crush, Retinal ganglion cell, Optic nerve, Neuroprotection

## Abstract

**Background:**

Optic neuropathy is a major cause of irreversible blindness, yet the molecular determinants that contribute to neuronal demise have not been fully elucidated. Several studies have identified ‘ephrin signaling’ as one of the most dysregulated pathways in the early pathophysiology of optic neuropathy with varied etiologies. Developmentally, gradients in ephrin signaling coordinate retinotopic mapping via repulsive modulation of cytoskeletal dynamics in neuronal membranes. Little is known about the role ephrin signaling plays in the post-natal visual system and its correlation with the onset of optic neuropathy.

**Methods:**

Postnatal mouse retinas were collected for mass spectrometry analysis for erythropoietin-producing human hepatocellular (Eph) receptors. Optic nerve crush (ONC) model was employed to induce optic neuropathy, and proteomic changes during the acute phase of neuropathic onset were analyzed. Confocal and super-resolution microscopy determined the cellular localization of activated Eph receptors after ONC injury. Eph receptor inhibitors assessed the neuroprotective effect of ephrin signaling modulation.

**Results:**

Mass spectrometry revealed expression of seven Eph receptors (EphA2, A4, A5, B1, B2, B3, and B6) in postnatal mouse retinal tissue. Immunoblotting analysis indicated a significant increase in phosphorylation of these Eph receptors 48 h after ONC. Confocal microscopy demonstrated the presence of both subclasses of Eph receptors within the retina. Stochastic optical reconstruction microscopy (STORM) super-resolution imaging combined with optimal transport colocalization analysis revealed a significant co-localization of activated Eph receptors with injured neuronal cells, compared to uninjured neuronal and/or injured glial cells, 48 h post-ONC. Eph receptor inhibitors displayed notable neuroprotective effects for retinal ganglion cells (RGCs) after six days of ONC injury.

**Conclusions:**

Our findings demonstrate the functional presence of diverse Eph receptors in the postnatal mammalian retina, capable of modulating multiple biological processes. Pan-Eph receptor activation contributes to the onset of neuropathy in optic neuropathies, with preferential activation of Eph receptors on neuronal processes in the inner retina following optic nerve injury. Notably, Eph receptor activation precedes neuronal loss. We observed a neuroprotective effect on RGCs upon inhibiting Eph receptors. Our study highlights the importance of investigating this repulsive pathway in early optic neuropathies and provides a comprehensive characterization of the receptors present in the developed retina of mice, relevant to both homeostasis and disease processes.

**Supplementary Information:**

The online version contains supplementary material available at 10.1186/s40662-023-00359-w.

## Introduction

Neuropathic diseases of the retina are a leading cause of irreversible blindness worldwide [1]. While many risk factors associated with the development of neuropathic diseases are known, the molecular determinants for the onset of synaptic instability, neurite retraction, and subsequent neuronal loss are yet to be fully elucidated. Identification of appropriate molecular targets is a crucial step in the development of effective therapies that can halt or reverse neuropathic progression and preserve useful sight. Pan-genomic and pan-proteomic profiling of glaucomatous and traumatic optic neuropathies has allowed for the construction of the first-ever roadmaps for system-wide characterization at the molecular level for the pathophysiology of common neuropathic mechanisms [2–5]. Strikingly, most of these studies have revealed that ‘ephrin signaling’ is one of the most dysregulated signaling cascades in the neurodegenerative process. Studies have further shown that signaling via activated erythropoietin-producing human hepatocellular (Eph) receptors in early neuropathic states is evident in both animal models and human samples. Eph receptors and ephrin ligands (efn) constitute the largest family of receptor tyrosine kinases in mammalian biology. To date, 16 Eph receptors have been identified and divided into subfamily A and subfamily B based on their sequence homologies and binding affinities [6–10].

Developmentally, Eph/efn signaling plays an important role in axon guidance and topographic mapping of neuronal projections [11–16], synaptogenesis and dendritic spine morphology [17–24], and synaptic plasticity and remodeling [19, 25–27]. Eph/efn signaling is distinctive in that its signal is transduced bidirectionally, with receptor–ligand interactions initiating signaling cascades in both receptor-expressing (forward) as well as ligand-expressing (reverse) cells simultaneously. Reverse signaling (efn-mediated) is generally regarded as an attractive and stabilizing stimulus for neuronal extensions and stabilizing synaptic connections such as ephrin-B3 [28, 29], whereas activated forward signaling (Eph-mediated) is repulsive to outgrowing neurites (EphA3, EphA4, EphA5, and EphB2) [7, 30] and is responsible for inducing axonal growth cone collapse (EphA4, EphA5, EphB1, EphB2, and EphB3) [31–37], restricting mid-line crossing of axons (EphA4, EphB1, EphB2, and EphB3) [38–42], and destabilizing synaptic connections (EphA1, EphA4, EphB1, EphB2, and EphB3) [42–46]. During development, Eph/efn signaling is responsible for establishing guiding gradients that direct the retinotopic projections of retinal ganglion cell (RGC) axons onto the visual centers of the brain [11–14, 47–51]. This guidance for the outgrowing RGC neurites established by Eph/efn signaling is achieved through graded repulsion rather than attraction [7, 31, 32, 38]. The upregulation of developmentally relevant programs in adult tissue is generally associated with mechanisms for repair and healing. However, the anachronic activation of growth repulsive pathways such as Eph forward signaling could result in detrimental outcomes. In fact, Eph receptor engagement and activation has been associated with several neurodegenerative disease states of the central nervous system including Alzheimer’s disease (EphA1, EphA4, EphA5, and EphB1) [44, 45, 52–55], glaucomatous degeneration of the retina (EphA2, EphB1, and EphB2) [2–5, 56–61], traumatic brain injury (EphA4, EphA6, and EphB3) [62–64], stroke (EphA4 and EphB2) [65–67], and spinal cord injury (EphA4, EphA7, and EphB2) [68–71].

The central nervous system lacks the ability to undergo endogenous repair, and given the repulsive nature of Eph receptor signaling, it is imperative to determine the role of these repulsive guidance programs in the neurodegenerative process. To this end, the identification of the molecular targets involved is the first step towards developing effective therapies for these diseases. In this study, we identified the Eph receptors expressed in the postnatal mouse retina, evaluated their early temporal expression and activation after injury, and determined their localization within the retinal layers and in specific cellular compartments following injury. We advance the hypothesis that neuropathic onset involves, at least in part, the reactivation of repulsive ephrin forward signaling on retinal neuronal membranes based on our findings. These results provide a rationale to evaluate modulation of this signaling pathway as a novel treatment for the management of optic neuropathies.

## Methods

### Optic nerve crush model (ONC)

All experiments involving mice were carried out in accordance with the ARVO statement for the Use of Animals in Ophthalmic and Vision research and were approved by the Animal Care and Use Committee at the University of Miami (IACUC animal protocol #21-122). ONC injury was performed on either male C57BL/6J WT or male Thy1-GFP mice at two months of age. The ONC procedure was performed as previously described [72]. Briefly, all mice were anesthetized via intraperitoneal injection of ketamine (80 mg/kg)/xylazine (10 mg/kg); their optic nerves were exposed intraorbitally and crushed with jeweler’s forceps (Dumon #5; tip dimension, 0.1 × 0.6 mm) for 10 s, approximately 1 to 2 mm behind the optic disk. Pupillary response using indirect illumination from the side was used to indicate a successful injury. Animals were allowed to recover from anesthesia and maintained in standard housing conditions for the duration of the specified experimental time points. Animals were euthanized and perfused with 1 × phosphate buffered saline (PBS) (Corning, Cat.# 21-040-CV), after which the eyes were enucleated, and the retinas micro-dissected. Dissected retinas were either fixed in 4% formaldehyde (MilliporeSigma, Cat.# FX0415-4) in PBS for 2 h at room temperature or lysed with RIPA buffer (Thermo Fisher Scientific, Cat.# 89900) containing protease and phosphatase inhibitors (Thermo Fisher Scientific, Cat.# A32961) depending on downstream analysis requirements.

### Mass spectrometry

A single proteomic analysis was done on dissected retinal tissue of male C57BL/6J WT mice at 14 days (*N* = 5 biological replicates, pooled), two months (*N* = 5 biological replicates), and 12 months of age (*N* = 4 biological replicates, pooled). Samples were processed and analyzed by solution digestion and 90-min data-independent acquisition by MS Bioworks Protein Mass Spectrometry Service, Ann Arbor, MI, USA. Briefly, flash-frozen dissected retinal tissue was lysed in a modified RIPA buffer (50 mM Trish HCl, pH 8.0, 150 mM NaCl, 2.0% SDS, 0.1% TX100, 1 × Roche Complete Protease Inhibitor) using 1.4 mm stainless steel beads in a Next Advance Bullet Blender, 2 cycles × 3 min each. Samples were then heated to 60 °C for 30 min and centrifuged at 16,000×*g*. Each sample was then TCA precipitated overnight at − 20 °C, pellets were washed and resuspended in 8 M urea, 50 mM Tris HCL, pH 8.0, and 1 × Roche Complete Protease Inhibitor. An equal aliquot of each pooled sample (14 days, 2 months, and 12 months) was taken to create a combined sample. 50 mg of the combined sample was digested overnight with trypsin. Samples were reduced for 1 h at room temperature in 12 mM DTT followed by alkylation for 1 h at room temperature in 15 mM iodoacetamide. Trypsin was added to an enzyme:substrate ratio of 1:20. Each sample was acidified to 0.3% TFA and subjected to SPE using Waters mHLB.

DIA chromatogram library generation was done using 1 mg of the pool and analyzed by nano LC/MS with a Water M-class HPLC system interfaced to a ThermoFisher Exploris 480. Peptides were loaded on a trapping column and eluted over a 75 mm analytical column at 350 nL/min; both columns were packed with XSelect CSH C18 resin; the trapping column contained a 5 mm particle; the analytical column contained a 2.4 mm particle. The column was heated to 55 °C using a column heater. A 90-min gradient was employed. The mass spectrometer was operated in data-independent mode. Six gas-phase fraction injections were acquired for six ranges: 396 to 502, 496 to 602, 596 to 702, 696 to 802, 796 to 902, and 896 to 1002. Sequentially, full-scan MS data (60.000 FWHM resolution) was followed by 26 × 4 m/z precursor isolation windows, another full-scan, and 26 × 4 m/z windows staggered by 2 m/z; products were acquired at 30,000 FWHM resolution. The automatic gain control target was set to 1 × 10^6^ for both full MS and product ion data. The maximum ion inject time was set to 50 ms for full MS and “dynamic” mode for products with nine data points required across the peak; the NCE was set to 30.

1 mg per sample was injected at random and analyzed by nano LC/MS with a Waters M-class HPLC system interfaced to a ThermoFisher Exploris 480. Peptides were loaded on a trapping column and eluted over a 75 mm analytical column at 350 nL/min; both columns were packed with XSelect CSH C18 resin; the trapping column contained a 5-mm particle; the analytical column contained a 2.4-mm particle. The column was heated to 55 °C using a column heater. A 90-min gradient was employed. The mass spectrometer was operated in data-independent mode. Sequentially, full-scan MS data (60,000 FWHM resolution) from m/z 385–1015 was followed by 61 × 10 m/z precursor isolation windows, another full-scan from m/z 385–1015 was followed by 61 × 10 m/z windows staggered by 5 m/z; products were acquired at 15,00 FWHM resolution. The maximum ion inject time was set to 50 ms for full MS and “dynamic” mode for products with nine data points required across the peak; the NCE was set to 30. An injection of the sample pool was included at the start, middle, and end of the batch. DIA data were analyzed using Scaffold DIA 3.2.1.

### Western blots

Retinal tissues (*N* = 3 biological replicates) were lysed with RIPA buffer (Thermo Fisher Scientific, Cat.# 89900) containing protease and phosphatase inhibitors (Thermo Fisher Scientific, Cat.# A32961), and protein concentrations were measured using the DC Assay (Bio-Rad Laboratories, Cat.# 5000114), according to the manufacturer’s protocol. An equal amount of protein samples was loaded and separated on an SDS-PAGE 7.5% PROTEAN TGX Stain-Free gel (Bio-Rad, Cat.# 4568024), then transferred to 0.2 µm PVDF membranes (Bio-Rad Laboratories, Cat.# 1,704,156). The PVDF membranes were blocked in 5% Nonfat dry milk (Bio-Rad Laboratories, Cat.# 1,706,404) in Tris-buffered saline with 0.1% Tween 20 (TBST) (VWR, Cat.# K873-4L). Samples were probed with the primary antibodies listed in Additional file 1: Table S1 overnight in 5% bovine serum albumin (Gold Biotechnology, Cat.# A-420-100) in TBST. Blots were incubated with horseradish peroxidase-conjugated species-specific secondary antibodies listed in Additional file 1: Table S1. Proteins were visualized with an enhanced chemiluminescence substrate (Thermo Fisher Scientific, Cat.# 34095) and digitally imaged on a ChemiDoc MP Imaging System (Bio-Rad Laboratories). Quantification of the band intensity was carried out using the Image J Software (NIH, Bethesda, MD).

### Immunofluorescent staining for confocal microscopy and stochastic optical reconstruction microscopy (STORM)

Dissected ONC and uninjured WT retinal tissue from C57BL/6 J was fixed for 2 h at room temperature in 4% formaldehyde (MilliporeSigma, Cat. # FX0415-4) in PBS (Corning, Cat.# 21-040-CV). ONC and uninjured tissues were embedded in 4% low-melting agarose (IBI Scientific, Cat.# IB70051) in PBS and sectioned (50 µm) using a Leica VT1000 S Vibrating blade microtome (Leica Biosystems). The sectioned tissues were permeabilized for 20 min in PBS (Corning, Cat.# 21-040-CV) containing 0.3% Triton X-100 (Thermo Scientific, Cat.# 85111) and blocked for 1 h in PBS containing 10% normal donkey serum (Abcam, Cat.# ab7475). Primary antibodies (listed in Additional file 1: Table S3) in PBS solution were applied overnight at 4 °C. AlexaFlour secondary antibodies (listed in Additional file 1: Table S2) were applied for 2 h at room temperature. We used 4′, 6-diamidino-2-phenylindole (DAPI) (1 µg/mL; Bio-Rad, Cat.# 1351303) to counterstain for confocal microscopy. The sectioned tissues were mounted on microscope slides with ProLong Diamond antifade mountant (Thermo Fisher Scientific, Cat.# P36965).

Dissected ONC and uninjured retinal tissue from Thy1-GFP was fixed for 2 h at room temperature in 4% formaldehyde (MilliporeSigma, Cat.# FX0415-4) in PBS. Flat-mount preparations were mounted on microscope slides with ProLong Glass antifade mountant (Thermo Fisher Scientific, Cat.# P36982).

### Confocal microscopy imaging

Confocal imaging was performed on a Leica AOBS SP8 confocal microscope (Leica Microsystems, Exton, PA). A HC PL APO 40 × /1.30 OIL CS2 objective lens was used and imaged with a continuously adjustable galvo scanner. Fluorescence-labelled proteins were excited by 405, 488, and 561 nm lasers. Three conventional PMTs and one high sensitivity PMT (HyD) were utilized to capture optical signals. Image acquisition and processing were accomplished on LAS X software.

### Stochastic optical reconstruction microscopy (STORM) imaging

STORM imaging and processing TIRF Imaging experiments were done with a Nikon eclipse Ti2 inverted microscope equipped with Nikon Instruments (N-STORM). A 100 × TIRF objective 1.49NA lens was utilized and imaged using a Hamamatsu C11440 ORCA-flash CMOS 4.0 camera. Images were acquired sequentially 10,000 frames per filter channel at 20 ms time duration. Retinal tissue (*N* = 3 biological replicates) labeled with JF646 secondary (Additional file 1: Table S2) were excited with 90% laser power from a 647 nm laser and A568 secondary (Additional file 1: Table S2) labeled samples were excited with a 561 nm laser at 100% laser power. Nikon Nd2 files were separated and converted to tiff files per channel by a custom python script. STORM localization analysis was carried out with either the ImageJ thunderstorm plugin (1.3–2014-11–08) or WindSTORM MATLAB code. Data was fitted with a Gaussian PSF model using weighted least-squares estimation for the thunderstorm plugin.

### Optimal transport colocalization analysis (OTC)

Individual regions of interest (ROIs) were processed using the OTC package [73] in R 4.2.1 by taking five 64 × 64 random samples with a matching counterpart for the second channel (Additional file 1: Fig. S1). The OTC curves were then paired and compared using the Mann–Whitney U test.

### Intravitreal injection of Eph receptor inhibitors

Thy1-GFP mice were first anesthetized via intraperitoneal injection of ketamine (80 mg/kg)/xylazine (10 mg/kg). A quantity of 2 μL of Eph receptor inhibitor (Additional file 1: Table S3) was then injected into the temporal part of one eye via a glass micropipette inserted just behind the ora serrata (intravitreal injection). All small molecules were dissolved in 25% Dimethyl sulfoxide (DMSO) (Thermo Fisher Scientific, Cat.# D12345) in PBS (Corning, Cat.# 21-040-CV). All animals were treated immediately after ONC injury and again 48 h post-ONC injury. Intravitreal injections of Eph Receptor Inhibitor experiments were performed using a vehicle consisting of 25% DMSO in PBS.

### Sholl analysis

Individual RGC images taken from the confocal microscope were imported and analyzed in FIJI, ImageJ2 (Version: 2.9.0/1.53t) software with the neuroanatomy Sholl Analysis plug-in. The program creates concentric circles from 10 to 200 µm, each with a radius of 5 µm, starting from the soma and outwards towards the dendritic branching. Total length and branching were analyzed for each image [74, 75].

### Statistical analysis

One-way ANOVA and Mann–Whitney U tests were calculated using GraphPad Prism 9 (San Diego, CA), with a *P* value of less than 0.05 considered statistically significant.

## Results

### Eph receptor analysis in postnatal retinas

**Fig. 1:**
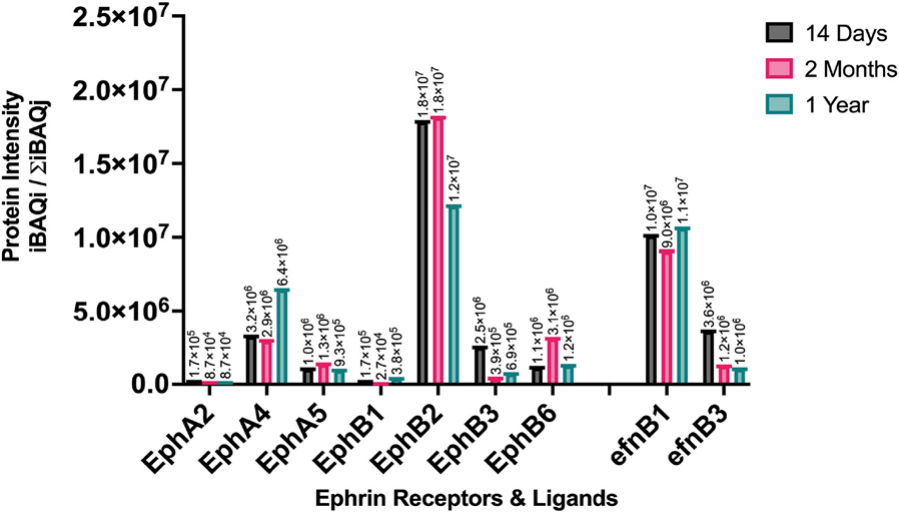
Proteomic analysis of dissected retinal tissue of male C57BL/6J WT mice at 14 days (*N* = 5 biological replicates, pooled), two months (*N* = 5 biological replicates, pooled), and 12 months of age (*N* = 5 biological replicates, pooled), using mass spectrometry data independent acquisition (MS-DIA). Temporal expression of Eph receptors and ephrin ligands in the postnatal retinas

Until now, it was unclear which of the many Eph receptors expressed during retinotopic development were still present in postnatal retinas. We used Mass Spectrometry Data Independent Acquisition (MS-DIA) to identify Eph receptors present in the uninjured postnatal retina in 14-day-old, two-month-old and one-year-old C57BL/6J mice. A single experiment analyzing pooled retinal tissue from five C57BL/6J mice for each respective time-point (*N* = 5 biological replicates, pooled). Our data showed that of the 16 known Eph receptors, three class-A receptors (EphA2, EphA4, and EphA5) and four class-B receptors (EphB1, EphB2, EphB3, and EphB6) are present in the postnatal mouse retinas (Fig. 1), and that their relative quantities remain unchanged throughout the lifespan of the animals. Of the expressed receptors, EphB2 and EphA4 were measured to be the most abundant, whereas EphA2 is the least common in all age groups (Fig. 1). Based on our MS-DIA data we narrowed our focus from the 16 known Eph receptors to the detected seven receptors in subsequent experiments.

### Examining Eph receptor expression and activation in normal and neuropathic retinal tissue

**Fig. 2:**
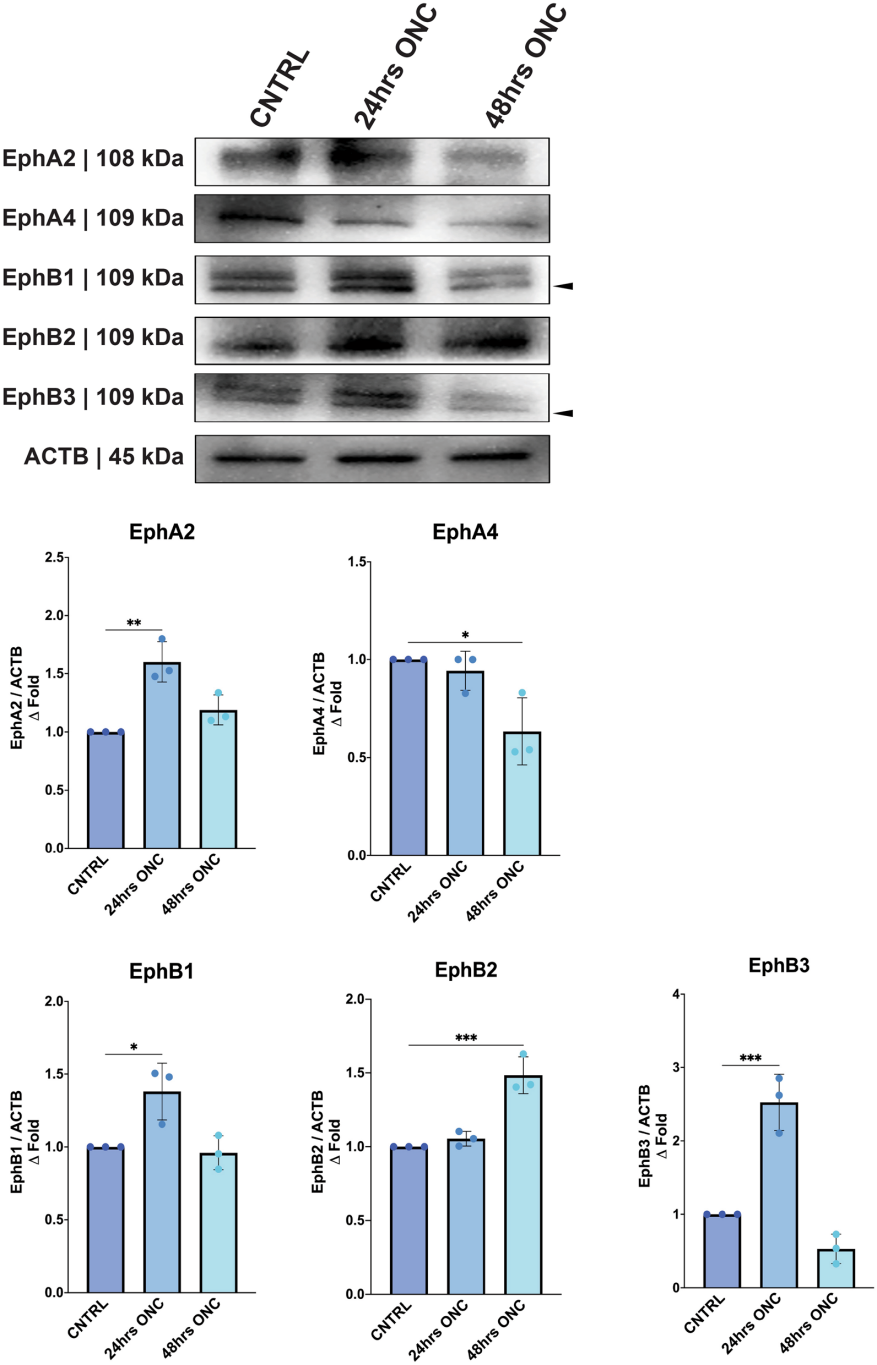
Proteomic quantification of Eph receptors 24 h and 48 h post optic nerve crush (ONC). Western blot detection and quantification of phosphorylated Eph receptors/b-actin from dissected whole retinal tissue 24 h and 48 h post-ONC. The geometric means and geometric standard deviations (*N* = 3 biological replicates) are graphed. A *P* value of less than 0.05 is considered statistically significant. *P ≤ 0.05, **P ≤ 0.01, ***P ≤ 0.001

Given the reported literature on ephrin signaling dysregulation in optic neuropathies, we speculated that Eph receptor activation would correlate with the onset of neuropathy and precede neuronal loss. We analyzed the proteomic changes in the retina elicited by ONC at 24- and 48-h post-injury (*N* = 3 biological replicates), when RGC loss is not yet significant in this model [76]. The proteomic analysis conducted on individual Eph receptors revealed substantial alterations in their expression profiles following injury. Specifically, EphA2 exhibited a statistically significant upregulation at the 24-h time point (*P* = 0.0019), while EphA4 demonstrated a significant increase 48 h post-injury (*P* = 0.0136). Similarly, EphB1 and EphB3 displayed significant upregulation in expression levels at 24 h post-injury (*P* = 0.0213 and *P* = 0.0006, respectively), while EphB2 exhibited increased expression 48 h after injury (*P* = 0.0005) (Fig. 2).

**Fig. 3:**
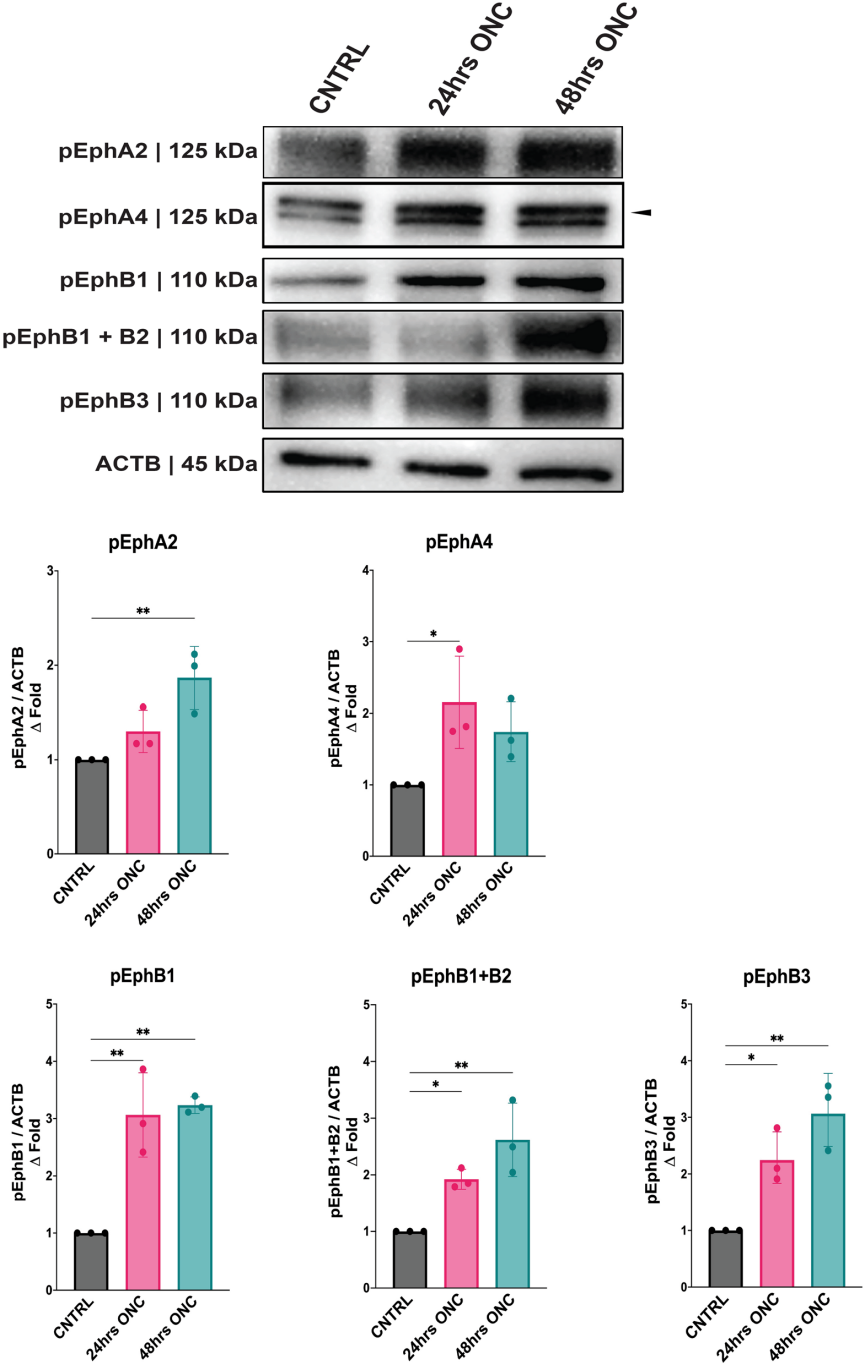
Proteomic quantification phosphorylated Eph receptors 24 h and 48 h post optic nerve crush (ONC). Western blot detection and quantification of Eph receptors/b-actin from dissected whole retinal tissue 24 h and 48 h post-ONC. The geometric means and geometric standard deviations (*N* = 3 biological replicates) are graphed. An arrow indicates the band of interest. A *P* value of less than 0.05 is considered statistically significant. *P ≤ 0.05, **P ≤ 0.01

Further proteomic analysis was conducted to investigate the impact of ONC-induced injury on Eph receptor signaling in the retina. The phosphorylated Eph receptors were analyzed at two time points, 24- and 48-h post-injury. The characterization and quantification of Eph receptor proteins revealed significant changes in their phosphorylation levels. Specifically, EphA2 showed a notable increase in phosphorylation at the 48-h mark (*P* = 0.0068). While EphA4 demonstrated significant activation at 24-h mark (*P* = 0.0331). EphB1 displayed a significant increase in phosphorylation at both 24 h (*P* = 0.0020) and 48 h (*P* = 0.0013) after the injury. Similarly, EphB3 exhibited a significant increase in phosphorylation at both 24 h (*P* = 0.0228) and 48 h (*P* = 0.0021) after the injury. Additionally, the use of a bivalent antibody targeting phosphorylated EphB1 + B2 detected a significant increase in phosphorylation at 24 h (*P* = 0.0468) and 48 h (*P* = 0.0039) post-injury (Fig. 3).

### Distribution of activated Eph receptors in the retinal layers following ONC and their association with neuronal and/or glial compartments

Global proteomic analysis and immunoblotting provides insights about the presence of Eph receptors and their relative phosphorylation states respectively, but they do not provide any information about where these receptors reside within the retina. To obtain this information, we used confocal as well as super-resolution microscopy to determine the location and cellular compartmentalization of activated Eph receptors following ONC injury. In our experiments, we used TUBB3 staining to identify neuronal cells and GLUL staining to label Müller glial cells, (*N* = 3 biological replicates).

**Fig. 4:**
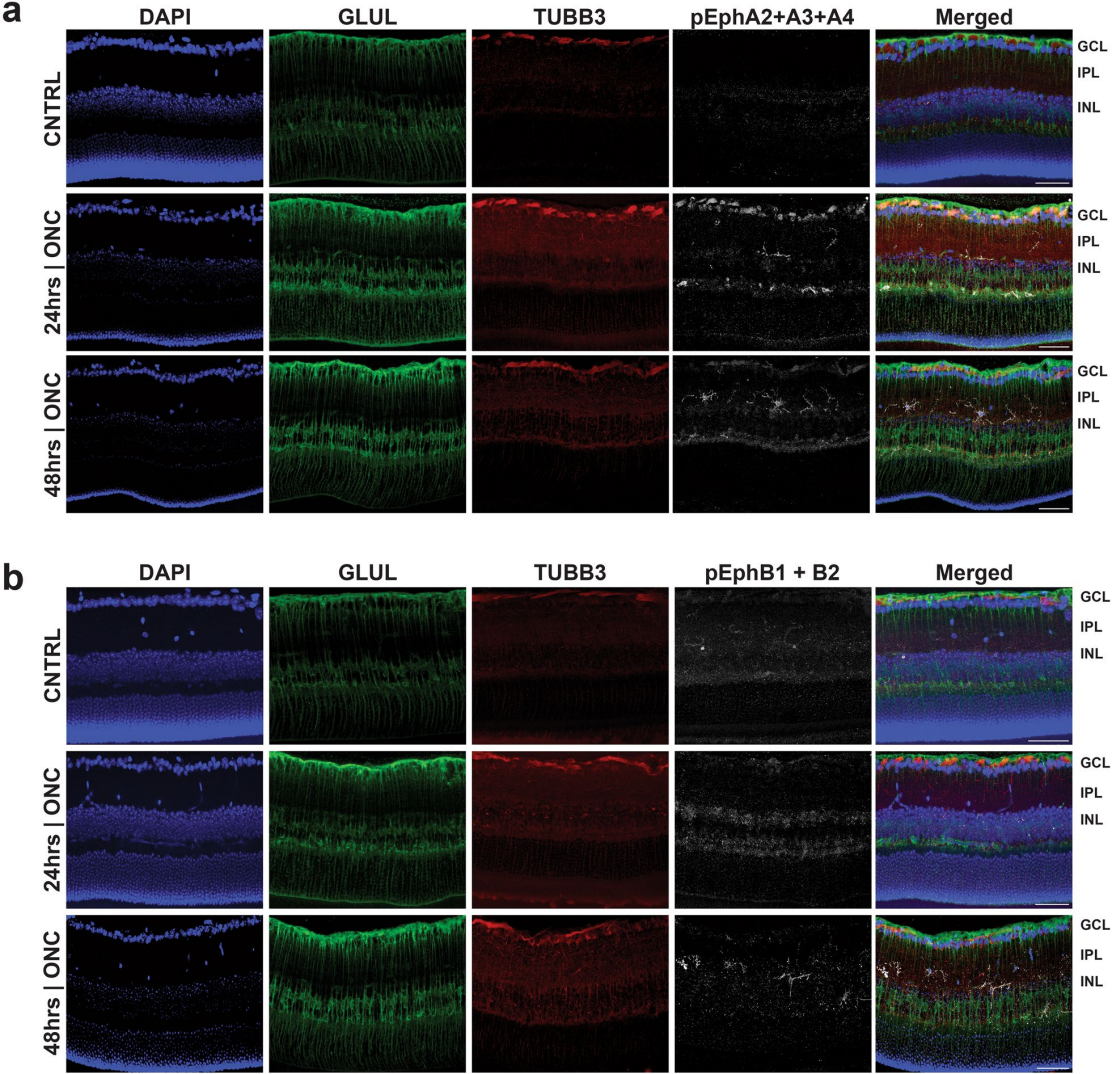
Phosphorylation of multiple EphA- and EphB-class receptors localized within the inner retina in early traumatic optic neuropathy. Immunofluorescent microscopy of retinas **a** Activated Eph receptors A2, A3, and A4, 24 h and 48 h post optic nerve crush (ONC); **b** Activated Eph receptors B1 and B2, 24 h and 48 h post-ONC. Scale bar at 50 μm. (*N* = 1 biological replicates), one biological replicate is depicted. CNTRL: uninjured control); DAPI, 4′,6-diamidino-2-phenylindole; GLUL, glutamine synthetase; TUBB3, Tubulin beta 3; GCL, ganglion cell layer; IPL, inner plexiform layer; INL, inner nuclear layer

Confocal microscopy imaging indicates that phosphorylated EphA2 + A3 + A4 are distributed extensively within the inner layers of the retinas (ganglion cell layer to inner plexiform layer) 24 and 48 h after ONC, and that the fluorescent intensity for these activated receptors is much higher in the injured retina than in non-injured controls at both time points (Fig. 4a). Similarly, phosphorylated EphB1 + B2 show a similar retinal layer distribution to the EphAs and are increased 24 h and more noticeably 48 h after ONC when compared to the uninjured retina (Fig. 4b).

**Fig. 5:**
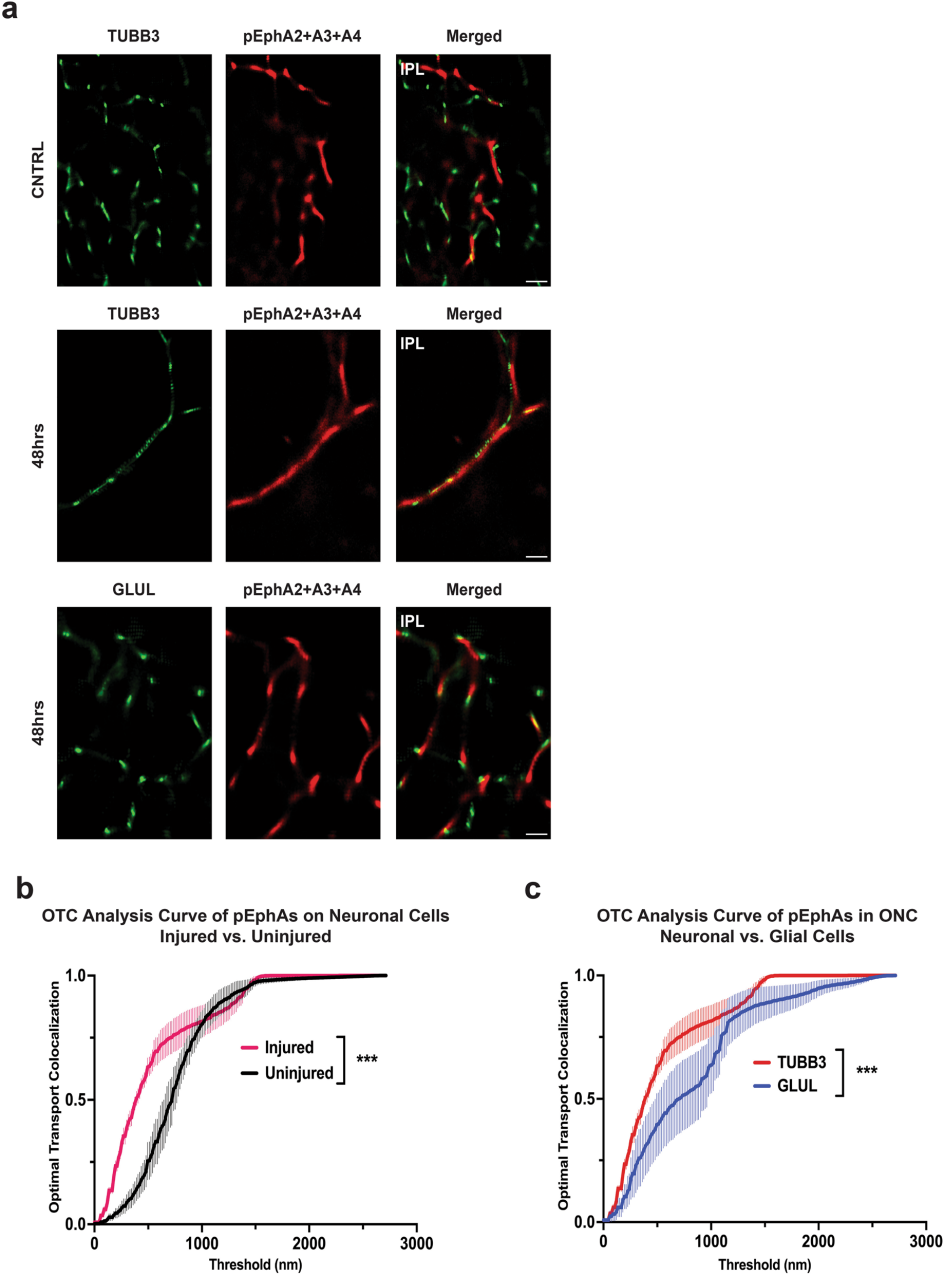
Super-resolution imaging and co-localization analysis of multiple EphA-class receptors in neuronal and glial cells within the inner plexiform layer (IPL) of 48 h optic nerve crush (ONC) retinas (injured). **a** Stochastic optical reconstruction microscopy (STORM) imaging of phosphorylated Eph receptors A2, A3, and A4 (pEphAs) in injured retinas. **b** Optical transport colocalization (OTC) analysis comparing the localization of pEphAs to neuronal cells (TUBB3) in uninjured and injured retinas. **c** OTC analysis comparing the localization of pEphBs to neuronal cells (TUBB3) and glial cells (GLUL) in injured retinas. *N* = 3 biological replicates are graphed; One biological replicate is depicted in the representative image. Mann–Whitney U tests and a *P* value of less than 0.05 is considered statistically significant. ***P ≤ 0.001. Scale bar at 10 μm

**Fig. 6:**
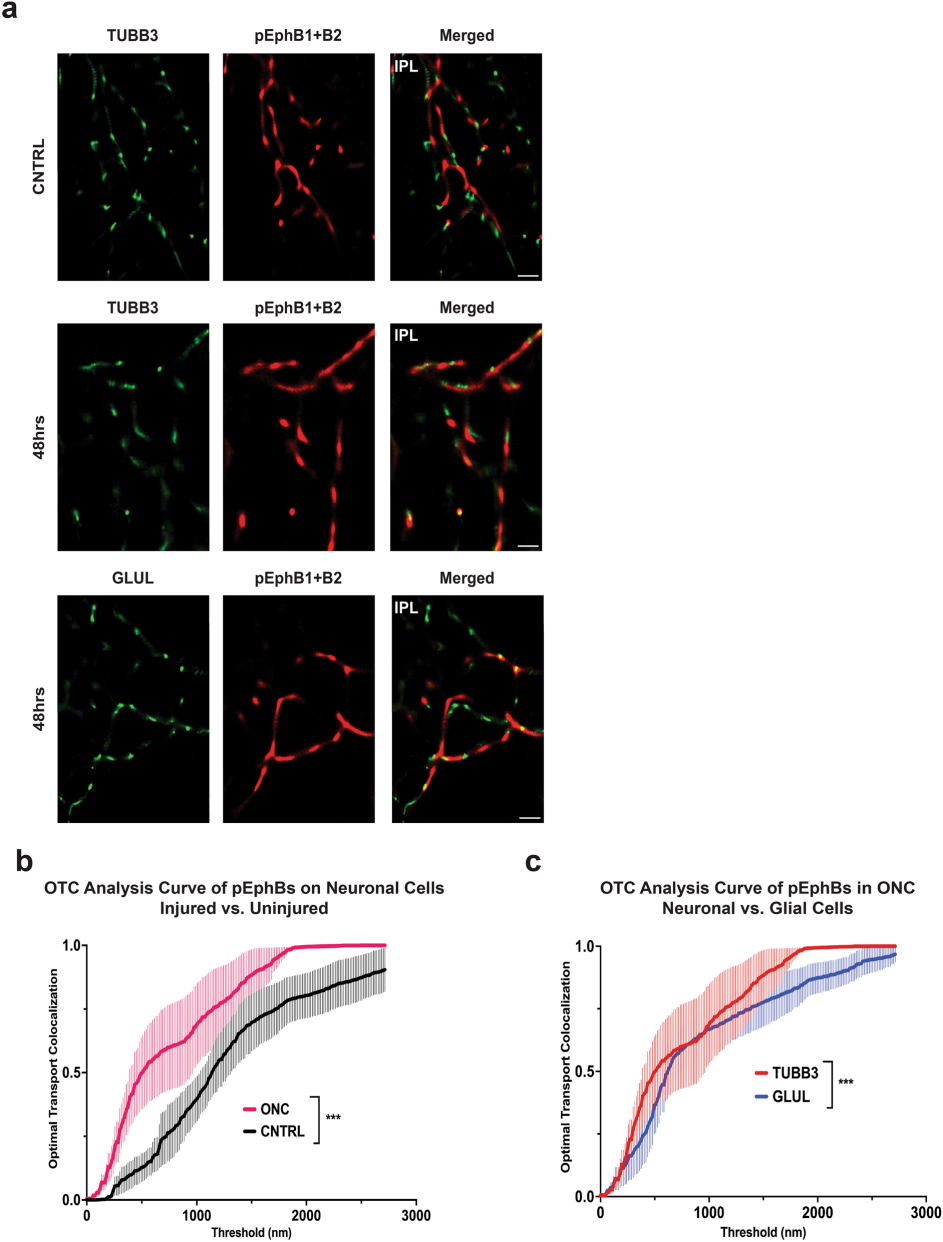
Super-resolution imaging and co-localization analysis of multiple EphB-class receptors in neuronal and glial cells within the inner plexiform layer (IPL) of 48 h optic nerve crush (ONC) retinas (injured). **a** Stochastic optical reconstruction microscopy (STORM) imaging of phosphorylated Eph receptors B1 and B2 (pEphBs) in injured retinas. **b** Optical transport colocalization (OTC) analysis comparing the localization of pEphBs to neuronal cells (TUBB3) in injured and uninjured retinas. **c** OTC analysis comparing the localization of pEphBs to neuronal cells (TUBB3) and glial cells (GLUL) in injured retinas. *N* = 3 biological replicates are graphed; One biological replicate is depicted in the representative image. Mann–Whitney U tests and a *P* value of less than 0.05 is considered statistically significant. ***P ≤ 0.001. Scale bar at 10 μm

STORM imaging followed by OTC co-localization analysis demonstrates that phosphorylated EphA2 + A3 + A4 at 48 h post-ONC are significantly more associated to injured neuronal cells than to uninjured neuronal cells (*P* = 0.003), and are significantly more associated to injured neuronal cells than to injured glial cells (*P* = 0.0001) (Fig. 5a–c). Similarly, STORM imaging and OTC co-localization analysis show that phosphorylated EphB1 + B2 are significantly more co-localized with injured neuronal cells than with uninjured neuronal cells (*P* = 0.0001) and again with injured neuronal cells over injured glial cells (*P* = 0.0002) (Fig. 6a–c).

### Neuroprotective effect of Eph receptor inhibition

The neuropathic progression is characterized by synaptic instability, retraction, and eventual dendritic and neuronal loss. Our evidence suggests that this process may be initiated through the activation of repulsive Eph receptor forward signaling on neuronal cells. However, the specific contributions of different classes of Eph receptors in this pathological mechanism remain unexplored. Therefore, it is essential to assess which classes may exert a more pronounced role in contributing the neuropathic degeneration of the retina. To evaluate this, we employed class specific Eph receptor inhibitors to preserve dendritic spines and arbor morphology in the context of neuropathic disease. Evaluating the individual dendritic arbor morphology of RGCs necessitates the sparse and selective labeling of individual RGCs, enabling accurate capture and prospective imaging of arbor morphology.

**Fig. 7:**
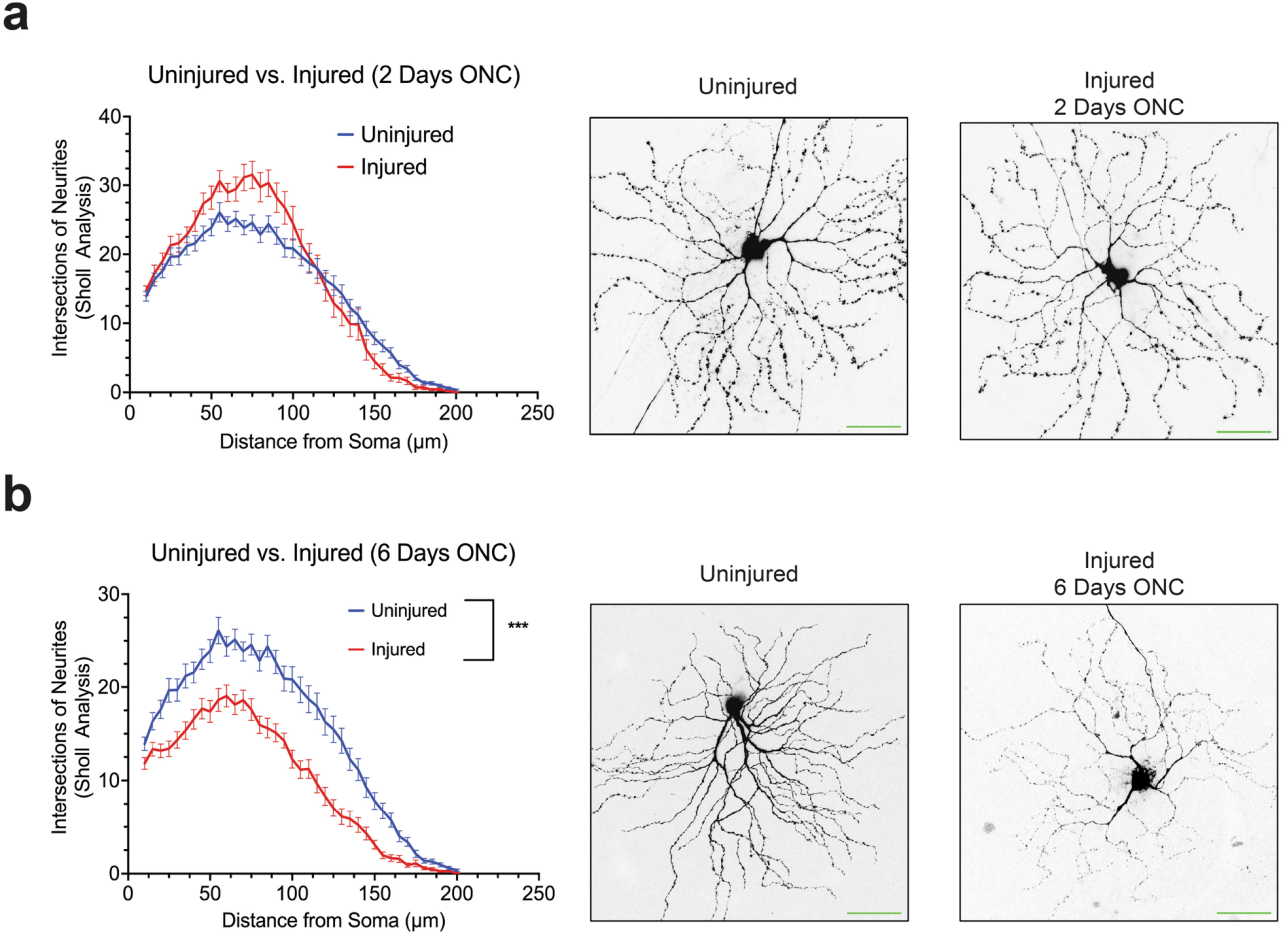
The dynamic change in retinal ganglion cell dendritic arborization detected by confocal microscopy post optic nerve crush (ONC) injury. **a** Quantification of retinal ganglion cell dendritic arborization by Sholl analysis two days after ONC injury (*N* = 3 biological replicates). **b** Quantification of retinal ganglion cell dendritic arborization by Sholl analysis six days after ONC injury (*N* = 3 biological replicates are graphed; One biological replicate is depicted in the representative image). Mann–Whitney U tests and a *P* value of less than 0.05 is considered statistically significant. ***P ≤ 0.001. Scale bar at 50 μm

To achieve this, we utilized two-month-old Thy1-GFP mice, a well-established transgenic mouse model featuring sparsely labeled fluorescence RGCs [75, 77, 78], in our ONC model. We examined the dendritic arbor morphology of Thy1-GFP mice at 48 h post-injury, a time point when RGC loss is not significant in this model [76], as well as at six days post-injury, when significant RGC loss occurs (*N* = 3 biological replicates). Our findings indicate that at 48 h post-injury, there were no significant changes in the dendritic arbor morphology of RGCs (*P* = 0.4942), consistent with previous literature [76] (Fig. 7a). Strikingly, at six days post-injury, a significant decrease in RGC dendritic arbor morphology was observed (*P* = 0.0017), in line with the literature [76] (Fig. 7b). These results validate the use of this in vivo model as a platform to evaluate the potential neuroprotective effect of Eph receptor inhibitors.

**Fig. 8:**
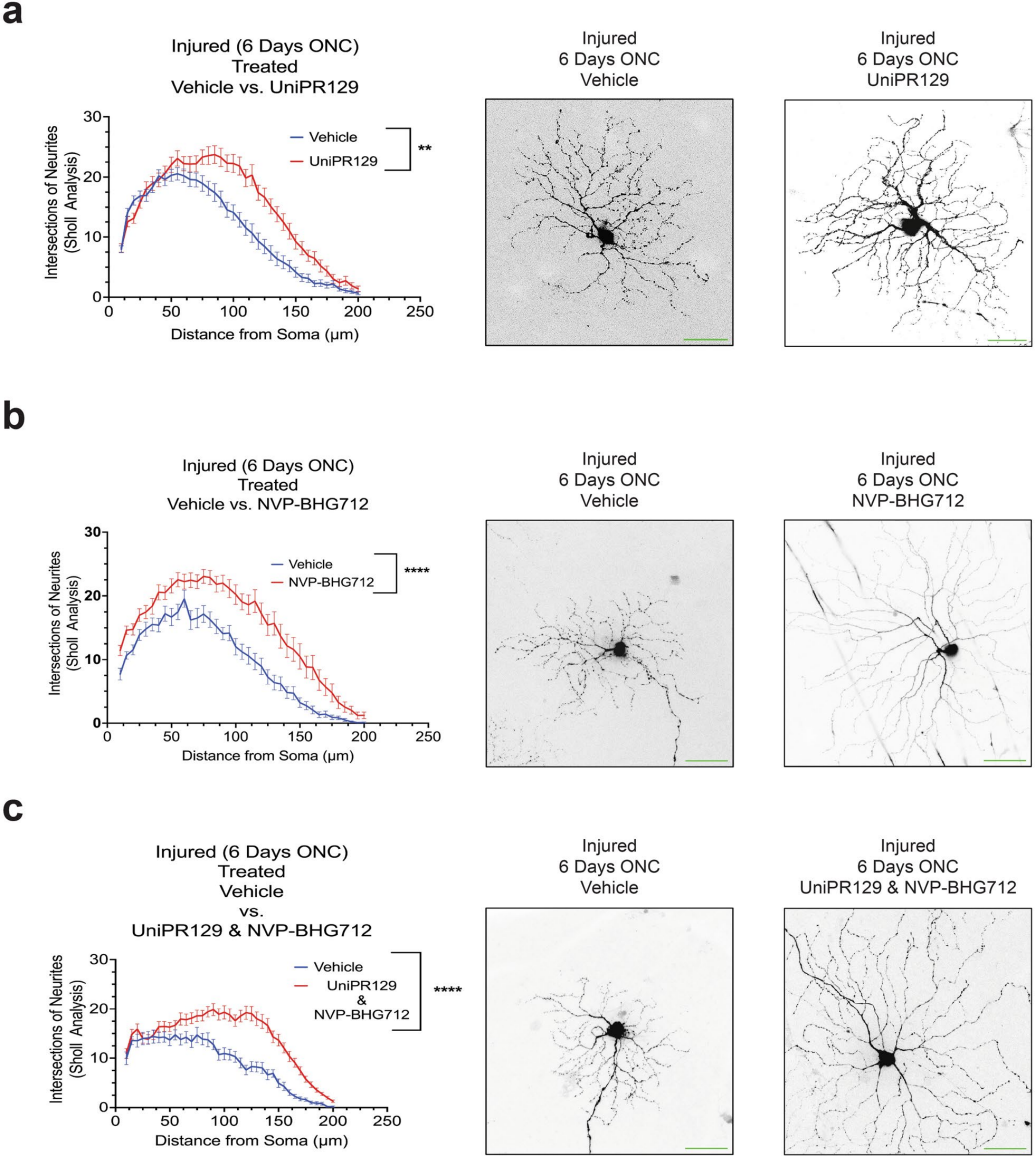
Pan Eph receptor inhibitors demonstrate neuroprotective properties, preserving retinal ganglion cell dendritic arborization as observed by confocal microscopy following six days post optic nerve crush (ONC) injury. **a** Quantification of retinal ganglion cell dendritic arborization by Sholl analysis six days after ONC injury. Animals treated with 50 μM of UniPR129 (*N* = 3 biological replicates). 25% DMSO in phosphate buffered saline (PBS) was used as a vehicle control. **b** Quantification of retinal ganglion cell dendritic arborization by Sholl analysis six days after ONC injury. Animals treated with 2 μM of NVP-BHG712 (*N* = 3 biological replicates). 25% DMSO in PBS was used as a vehicle control. **c** Quantification of retinal ganglion cell dendritic arborization by Sholl analysis six days after ONC injury. Animals treated with a combination of both UniPR129 (50 μM) and NVP-BHG712 (2 μM) (*N* = 3 biological replicates are graphed; One biological replicate is depicted in the representative image). Mann–Whitney U tests and a *P* value of less than 0.05 is considered statistically significant. **P ≤ 0.01, ****P ≤ 0.0001. Scale bar at 50 μm

We have shown a correlation between the activation of different Eph receptors and the retraction of neurites in RGCs. However, there is currently a lack of pan-Eph receptor inhibitors available in the market. Although specific inhibitors targeting individual Eph receptors do exist, the potential neuroprotective effect of Eph receptor inhibition using commercially available agents, administered intravitreally, remains to be explored. This experiment aimed to evaluate the neuroprotective potential of Eph receptor inhibition on preserving RGC dendritic arborization six days after injury (*N* = 3 biological replicates). The Eph receptor A preferential inhibitor UniPR129 (50 μM in 25% DMSO in PBS) [79] exhibited a significant neuroprotective effect (*P* = 0.0126) (Fig. 8a), while the Eph receptor B preferential inhibitor NVP-BHG712 (2 μM in 25% DMSO in PBS) [80] displayed a greater neuroprotective effect (*P* = 0.0004) (Fig. 8b). Strikingly, the most substantial neuroprotection was observed with the combination of both Eph receptor A and B preferential inhibitors (Fig. 8c). No toxic effects of the vehicle were observed.

## Discussion

Central neurodegeneration is a complex and multifactorial process [81–83]. Identifying the molecular determinants underlying the onset and progression of neuropathic states is fundamental to the development of effective treatments. Several lines of evidence show that ‘ephrin signaling’ is one of the most dysregulated pathways in optic neuropathies with varied etiologies [2–5].

Ephrin signaling is highly relevant to the visual system and retina, as counter-gradients of the various members in this ligand-receptor family mediate the establishment of the dorso-ventral and naso-temporal axes during retinotopic map formation in the retina and superior colliculus, as well as guide the decussation of RGC projections through the optic chiasm [11, 13, 14, 47, 49–51, 84–86]. Ephrin signaling through Eph receptors on RGC projections achieve their guidance by exerting a graded repulsive stimulus on the actin cytoskeletal dynamics across the neuronal cells [7, 31, 32, 38]. It is intriguing to observe then, that this developmental pathway would be so prominently dysregulated in neuropathic diseases of the visual system. While it can be proposed that the dysregulation constitutes a reactive response by the system in its attempt to re-establish retinotopy, it is interesting to speculate that, given its repulsive and destabilizing nature, activation of the Eph forward signaling pathway may ultimately contribute to neuropathic onset or progression. This study aimed to advance the understanding of dysregulated Eph receptor signaling during early optic neuropathies by addressing key unanswered questions. Firstly, we sought to identify the specific members of this extensive receptor tyrosine kinase family that persist in the postnatal retina. Secondly, we aimed to determine which of these receptors are engaged and dysregulated during neuropathic onset. Lastly, we aimed to elucidate the localization of these receptors and their presence on different cellular compartments within the retina. Previous studies have demonstrated the potential regenerative effects in the central nervous system, particularly the visual system, through modulation of specific Eph receptors [87–89]. Notably, multiple Eph family members have been implicated in the onset and progression of retinal neuropathic diseases [35, 38]. This is unsurprising considering the involvement of nearly all Eph receptors in the development of retinotopic projections. Considering this, our study aimed to thoroughly investigate the roles of both EphA- and EphB-class receptors using well-established small molecules that exhibit a preferential class-specific antagonism. Specifically, we utilized UniPR129 as an inhibitor for EphA-class receptors [79], and NVP-BHG712 as an inhibitor for EphB-class receptors [80]. These inhibitors have been shown in previous studies to selectively block their respective classes of Eph receptors, with minimal cross-reactivity between the two classes, as documented in published data [79, 80].

Our results show that at least seven Eph receptors remain patent in the post-natal mouse retina, and that their relative abundance does not change throughout the lifespan of the animals. We further demonstrate that all available Eph receptors become hyperphosphorylated during early time points (24 h and 48 h) following optic nerve injury prior to when neuronal dropout has been shown to be significant [76]. We show that the distribution of Eph receptors is confined to inner retinal structures, from the ganglion cell layer to the inner plexiform layer (IPL) and that, within the IPL, activation of Eph receptors occurs on neuronal cells and not on glial processes upon injury. Finally, we demonstrate that inhibiting ephrin signaling exhibits a significant neuroprotective effect on RGCs when modulating class specific Eph receptors with the most substantial protection observed with the combination of both Eph receptor A and B preferential inhibitors.

Our study does not allow for the determination of the cause and modality of Eph receptor activation; however, we know that RGCs project their dendritic arbors into the IPL where they synapse with other neurons of the retina. The IPL is also the site for most glial-neuron interactions and synaptic modulation. Optic neuropathies have a strong neuroinflammatory component to their pathophysiology [90, 91], and the loss of synaptic spine density has been shown to precede RGC loss in both glaucoma and traumatic optic neuropathy models [76, 92]. Whether glial swelling during optic neuropathy is the cause of Eph receptor activation on RGCs, and whether that constitutes a viable therapeutic target are matters of ongoing investigation by our group. But consistent with this idea, earlier research has demonstrated that ablating an individual Eph receptor has a positive impact on visual function and recovery in optic neuropathy models [88].

In line with our findings, Joly et al. [88] demonstrated that selective deletion of the EphA4 receptor in RGCs, while leaving the ligand efnA3 unaffected, led to significant regeneration following ONC. Similarly, Vilallongue et al. [89] reported comparable regenerative effects by knocking down both EphB2 and EphA4, as evidenced by an increase in the number of regenerative events. Our study highlights the aberrant activation of multiple Eph receptors following optic nerve injury, consistent with previous findings reported by Zhou et al. [6], Himanen et al. [93], and Kania et al. [94]. These studies have provided insights into the redundancy and compensatory mechanisms within this highly conserved developmental pathway. Our findings support the hypothesis that comprehensive or broad modulation of Eph receptor activity could significantly enhance visual outcomes in optic neuropathy by effectively safeguarding the dendritic arborization of RGCs.

The current clinical management of optic neuropathy primarily aims to control disease-associated risk factors, such as elevated intraocular pressure, and slow down disease progression [95–97]. However, advancements in high-throughput assays, coupled with parallel computing and bioinformatics algorithms, now enable the generation of comprehensive system-wide profiles of disease processes and molecular targets. Multiple independent studies have identified dysregulated 'ephrin signaling' as a principal component of optic neuropathy pathobiology [3, 5]. Remarkably, dysregulation of Eph receptor signaling is detected prior to the onset of visual functional decline, suggesting its involvement in the disease's pathogenesis rather than being merely another risk factor. These observations hold significant importance and require further exploration to understand their role in neuropathic progression. Identifying appropriate molecular targets is crucial for developing effective therapies for these conditions. By focusing on active ephrin forward signaling as a molecular determinant of neuropathic progression in the visual system, we aim to establish a framework for novel treatments that preserve and restore vision in patients with these debilitating conditions.

## Conclusions

This study demonstrates that all detectable Eph receptors within the postnatal murine retina become aberrantly hyperactivated on neuronal cells within the inner plexiform layer in the acute phase of optic neuropathic onset, and prior to a significant decline in RGC numbers following ONC injury. Given the strong repulsive and destabilizing effect that Eph forward signaling exerts on neuronal cells, these results constitute a significant advance in our characterization of the molecular determinants of RGC deterioration, a fundamental component of neuropathic diseases of the visual system, underscoring the need to further elucidate the role that Eph receptor signaling plays in disease progression and its value as a therapeutic target.

### Supplementary Information

**Additional file 1: Figure S1.** Illustration of optimal transport colocalization curve analysis workflow. Created with BioRender.com. **Figure S2.** Proteomic depiction of Eph receptors and phosphorylated Eph receptors 24 h and 48 h post optic nerve crush (ONC). **a** Western blot detection of Eph receptors/b-actin from dissected whole retinal tissue 24 h and 48 h post-ONC. **b** Western blot detection of phosphorylated Eph receptors/b-actin from dissected whole retinal tissue 24 h and 48 h post ONC. **c** Western blot quantification of phosphorylated Eph receptors/ Eph receptors from dissected whole retinal tissue 24 h and 48 h post-ONC. The geometric means and geometric standard deviations (*N* = 3 biological replicates) are graphed; One biological replicate is depicted the representative image. A one-way ANOVA was applied with a *P* value of less than 0.05 considered statistically significant. *P ≤ 0.05, **P ≤ 0.01, ***P ≤ 0.001, ****P ≤ 0.0001. An arrow indicates the band of interest. **Table S1.** Antibodies used for Western blotting. **Table S2.** Antibodies used for immunofluorescence and STORM staining. **Table S3.** Eph receptor inhibitors used in intravitreal injections.

## Data Availability

The datasets supporting the conclusions of this article are available in the Mendeley Data repository, Strong, Tom (2023), “Strong 2023 Eph Receptors”, Mendeley Data, V1, https://doi.org/10.17632/zcpzvjb2vw.1

## Abbreviations

efn: Ephrin ligands
Eph: Erythropoietin-producing human hepatocellular
MS-DIA: Mass spectrometry data independent acquisition
ONC: Optic nerve crush
ON: Optic neuropathy
OTC: Optical transport colocalization
RGC: Retinal ganglion cell
STORM: Stochastic optical reconstruction microscopy
