# Supplementary material for: Activation of multiple Eph receptors on neuronal membranes correlates with the onset of optic neuropathy
Source: Eye Vis (Lond). 2023 Oct 2;10:42. doi: 10.1186/s40662-023-00359-w (PMC10544557; doi:10.1186/s40662-023-00359-w)
Supplement: Supplementary file 1 — Additional file 1: Figure S1. Illustration of optimal transport colocalization curve analysis workflow. Created with BioRender.com. Figure S2. Proteomic depiction of Eph receptors and phosphorylated Eph receptors 24 h and 48 h post optic nerve crush (ONC). a Western blot detection of Eph receptors/b-actin from dissected whole retinal tissue 24 h and 48 h post-ONC. b Western blot detection of phosphorylated Eph receptors/b-actin from dissected whole retinal tissue 24 h and 48 h post ONC. c Western blot quantification of phosphorylated Eph receptors/ Eph receptors from dissected whole retinal tissue 24 h and 48 h post-ONC. The geometric means and geometric standard deviations (N = 3 biological replicates) are graphed; One biological replicate is depicted the representative image. A one-way ANOVA was applied with a P value of less than 0.05 considered statistically significant. *P ≤ 0.05, **P ≤ 0.01, ***P ≤ 0.001, ****P ≤ 0.0001. An arrow indicates the band of interest. Table S1. Antibodies used for Western blotting. Table S2. Antibodies used for immunofluorescence and STORM staining. Table S3. Eph receptor inhibitors used in intravitreal injections. [file 40662_2023_359_MOESM1_ESM.docx]

**ADDITIONAL FILE**

Figure S1. Illustration of optimal transport colocalization curve analysis workflow. Created with BioRender.com


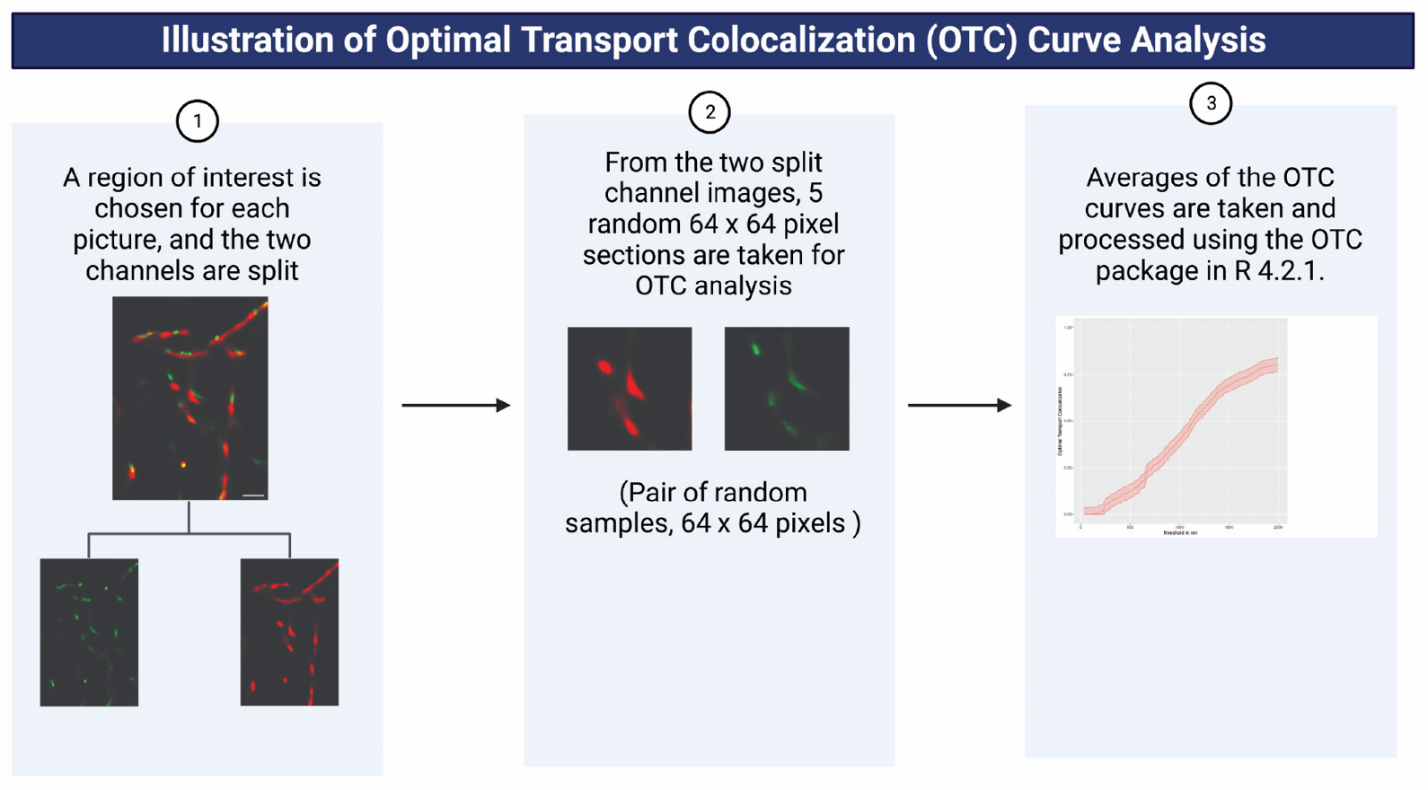


Figure S2. Proteomic depiction of Eph receptors and phosphorylated Eph receptors 24 hours and 48 hours post optic nerve crush (ONC). **a** Western blot detection of Eph receptors/b-actin from dissected whole retinal tissue 24 hours and 48 hours post ONC. **b** Western blot detection of phosphorylated Eph receptors/b-actin from dissected whole retinal tissue 24 hours and 48 hours post ONC. **c** Western blot quantification of phosphorylated Eph receptors/ Eph receptors from dissected whole retinal tissue 24 hours and 48 hours post ONC. The geometric means and geometric standard deviations (*N* = 3 biological replicates) are graphed; One biological replicate is depicted the representative image. A one-way ANOVA was applied with a *P* value of less than 0.05 considered statistically significant. **P* ≤ 0.05, ***P* ≤ 0.01, ****P* ≤ 0.001, *****P* ≤ 0.0001. An arrow indicates the band of interest.


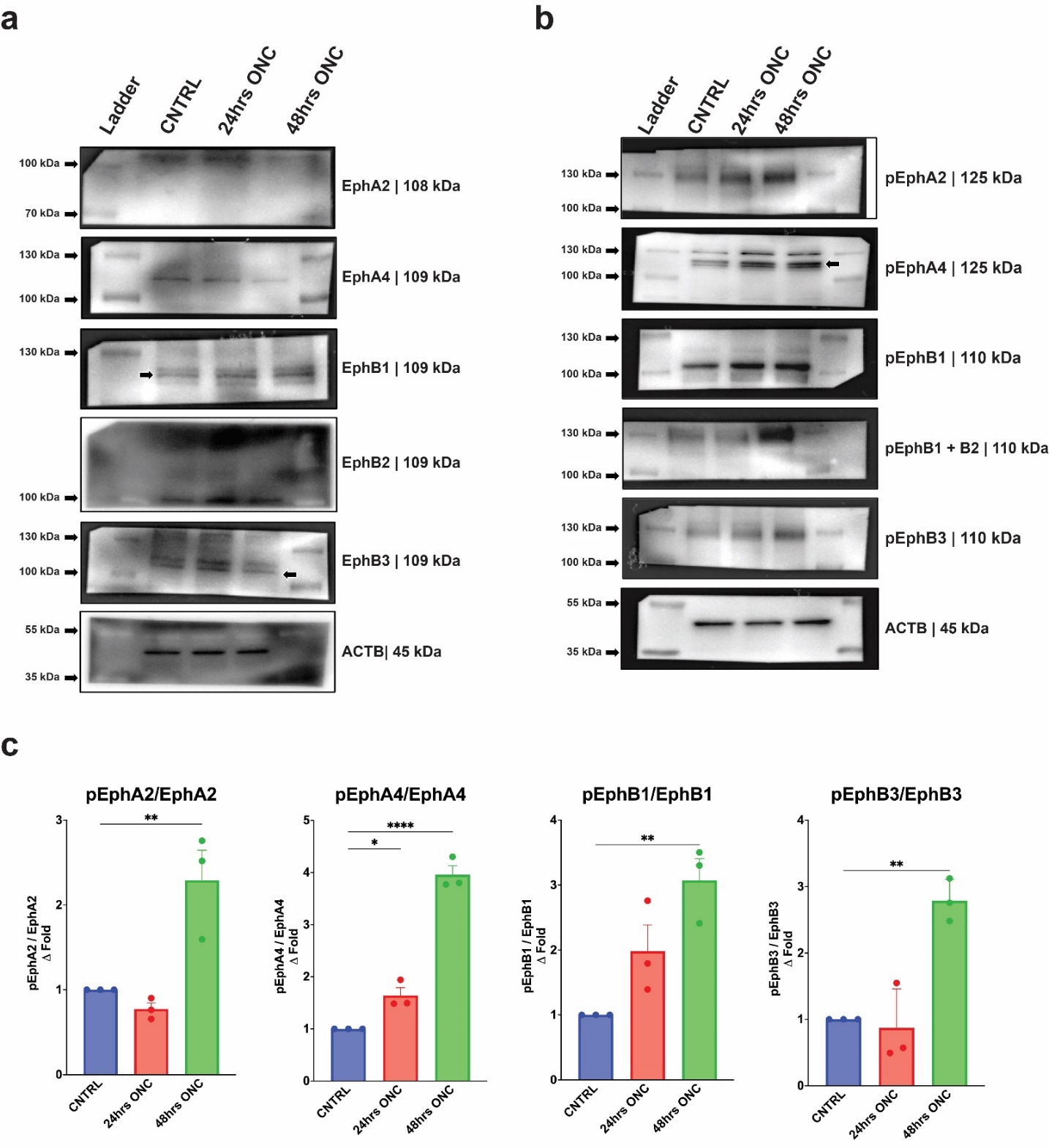


Table S1. Antibodies used for Western blotting

| Antibodies | Source | Cat# | Host | Dilution | RRID |
| --- | --- | --- | --- | --- | --- |
| Phospho-EphA2 | Cell Signaling Technology | 8244S | Rb | 1:500 | AB_10860415 |
| Phospho-EphA4 | Thermo Fisher Scientific | PA5-37562 | Rb | 1:500 | AB_2554170 |
| Phospho-EphA5 | Thermo Fisher Scientific | PA5-64786 | Rb | 1:500 | AB_2662013 |
| Phospho-EphB1 | abcam | ab129103 | Rb | 1:500 | AB_11142578 |
| Phospho-EphB1+B2 | abcam | ab61791 | Rb | 1:500 | AB_2099832 |
| Phospho-EphB3 | Thermo Fisher Scientific | PA5-106134 | Rb | 1:500 | AB_2817532 |
| EphA2 | Cell Signaling Technology | 6997S | Rb | 1:500 | AB_10827743 |
| EphA4 | abcam | ab5396 | Rb | 1:500 | AB_304857 |
| EphB1 | ThermoFisher Scientific | PA5-14604 | Rb | 1:500 | AB_2099828 |
| EphB2 | ThermoFisher Scientific | MA5-11162 | Ms | 1:500 | AB_10980840 |
| EphB3 | abcam | ab133742 | Rb | 1:500 | AB_2938587 |
| Beta Actin | Thermo Fisher Scientific | MA5-15739 | Ms | 1:1000 | AB_10979409 |
| Mouse IgG HRP-conjugated | R&D Systems | HAF007 | Gt | 1:1000 | AB_357234 |
| Rabbit IgG HRP-conjugated | R&D Systems | HAF008 | Gt | 1:1000 | AB_357235 |

Table S2. Antibodies used for immunofluorescence and STORM staining

| Antibodies | Source | Cat# | Host | Dilution | RRID |
| --- | --- | --- | --- | --- | --- |
| Phospho-EphA2+A3+A4 | abcam | ab62256 | Rb | 1:100 | AB_942240 |
| Phospho-EphB1+B2 | abcam | ab61791 | Rb | 1:100 | AB_2099832 |
| Anti-beta III Tubulin | Novus Biologicals | NB100-1612 | Ck | 1:100 | AB_10000548 |
| Anti-Glutamine Synthetase | Millipore Sigma | MAB302 | Ms | 1:100 | AB_2110656 |
| Alexa Fluor 488^IF^ | abcam | ab150113 | Gt | 1:100 | AB_2576208 |
| Alexa Fluor 555^IF^ | abcam | ab150170 | Gt | 1:100 | AB_2893330 |
| Alexa Fluor 647^IF^ | abcam | ab150079 | Gt | 1:100 | AB_2722623 |
| Janelia Fluor 646^STORM^ | Novus Biologicals | NBP1-72732JF646 | Gt | 1:100 | AB_2936900 |
| Alexa Fluor 568^STORM^ | ThermoFisher Scientific | A-11004 | Gt | 1:100 | AB_2534072 |

Table S3. Eph receptor inhibitors used in intravitreal injections

| Small Molecule | Source | Cat# | Injected Conc. | CID |
| --- | --- | --- | --- | --- |
| UniPR129 | MilliporeSigma | 533871 | 50 μM | 91668293 |
| NVP-BHG712 | abcam | ab61791 | 2 μM | 16747388 |
